# Supplementary material for: Population genomics and the evolution of virulence in the fungal pathogen Cryptococcus neoformans
Source: Genome Res. 2017 Jul;27(7):1207–19. doi: 10.1101/gr.218727.116 (PMC5495072; doi:10.1101/gr.218727.116)
Supplement: Supplemental Material [file supp_27_7_1207__index.html]

Population genomics and the evolution of virulence in the fungal pathogen Cryptococcus neoformans — Population genomics and the evolution of virulence in the fungal pathogen Cryptococcus neoformans — Supplemental Material 

# Population genomics and the evolution of virulence in the fungal pathogen *Cryptococcus neoformans*

## Supplemental Material

- Supplemental\_Fig\_S1.pdf
- Supplemental\_Fig\_S2.pdf
- Supplemental\_Fig\_S3.pdf
- Supplemental\_Fig\_S4.pdf
- Supplemental\_Fig\_S5.pdf
- Supplemental\_Fig\_S6.pdf
- Supplemental\_Fig\_S7.pdf
- Supplemental\_Fig\_S8.pdf
- Supplemental\_Methods.docx
- Supplemental\_Table\_S1.xlsx
- Supplemental\_Table\_S2.docx
- Supplemental\_Table\_S3.docx
- Supplemental\_Table\_S4.docx
- Supplemental\_Table\_S5.xlsx
- Supplemental\_Table\_S6.docx
- Supplemental\_Table\_S7.docx
- Supplemental\_Table\_S8.docx
- Supplemental\_Table\_S9.docx
- Supplemental\_Table\_S10.docx
- Supplemental\_Table\_S11.docx
- Supplemental\_Table\_S12.docx
