# Supplementary material for: Population genomics and the evolution of virulence in the fungal pathogen Cryptococcus neoformans
Source: Genome Res. 2017 Jul;27(7):1207–19. doi: 10.1101/gr.218727.116 (PMC5495072; doi:10.1101/gr.218727.116)
Supplement: Supplemental Material [file supp_gr.218727.116_Supplemental_Fig_S2.pdf]

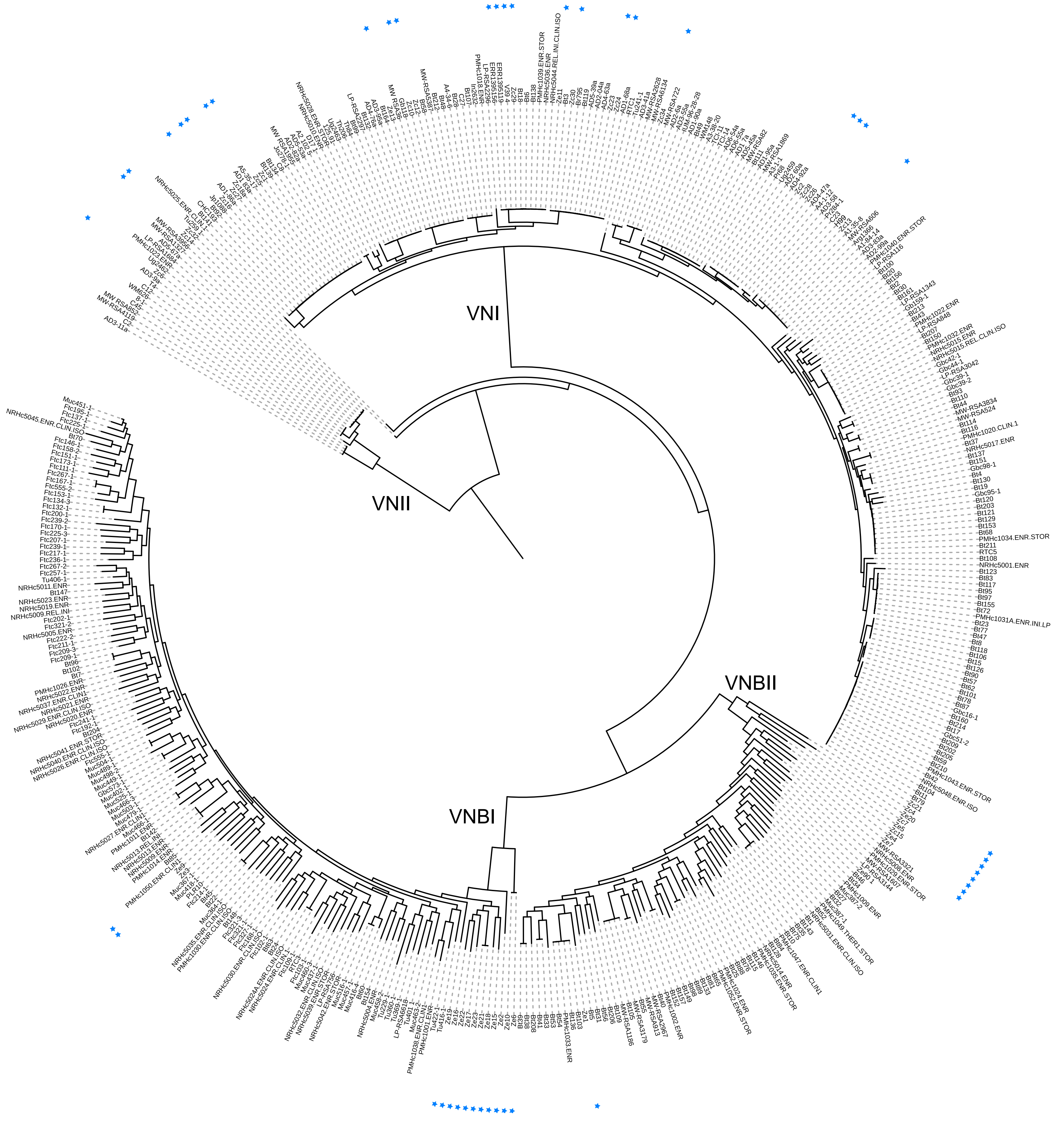

Supplemental Fig S2. Zambian isolates are distributed across the phylogenetic tree. A combine phylogeny was estimated from both the data in this study and previously published Zambian isolates (Vanhove et al. 2016). Zambian isolates are highlighted with blue stars. Zambian VNBI and VNBII isolates show ancestral diversity, while Zambian VNI isolates are scattered throughout global the global VNI phylogeny.
