# Supplementary material for: Population genomics and the evolution of virulence in the fungal pathogen Cryptococcus neoformans
Source: Genome Res. 2017 Jul;27(7):1207–19. doi: 10.1101/gr.218727.116 (PMC5495072; doi:10.1101/gr.218727.116)
Supplement: Supplemental Material [file supp_gr.218727.116_Supplemental_Fig_S3.pdf]

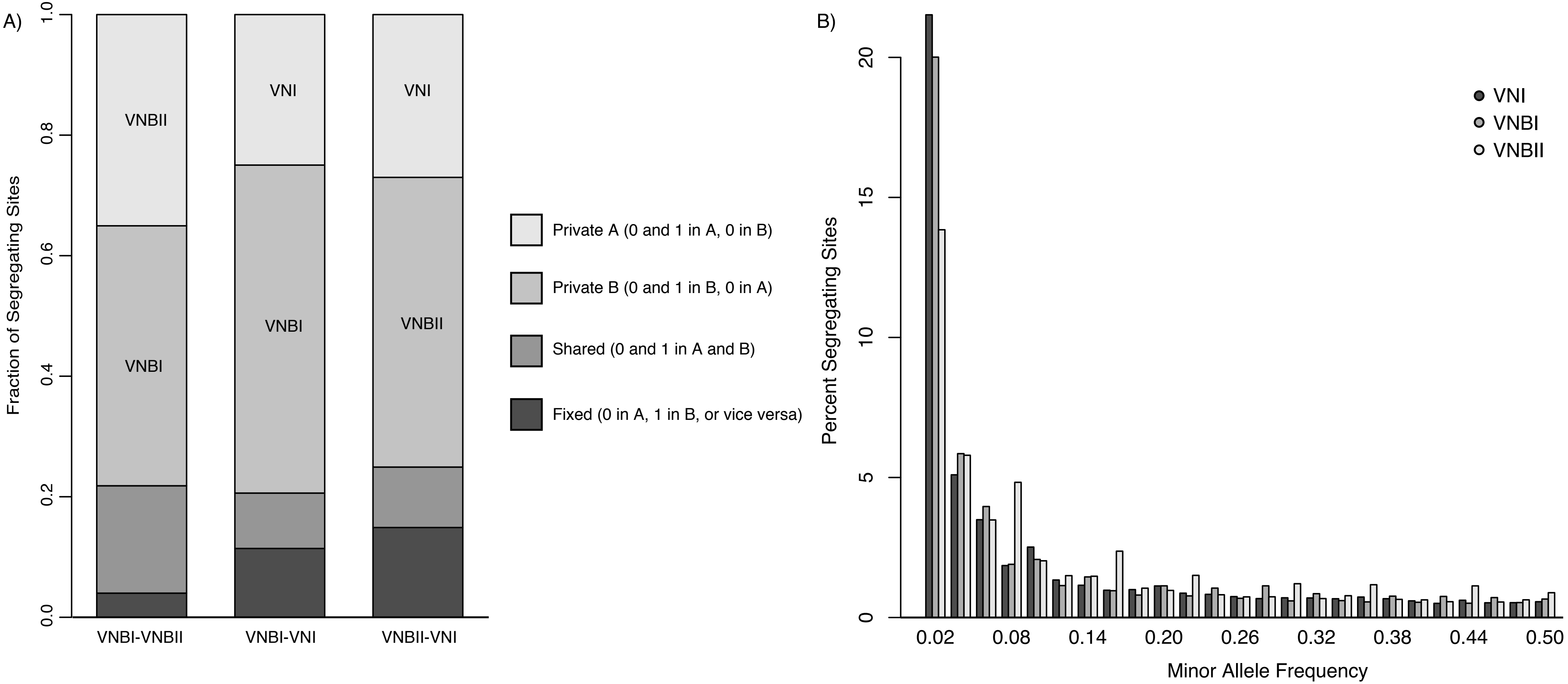

Supplemental Fig S3. Analysis of allele frequencies identified an excess of shared alleles between and private alleles within VNBI and VNBII, and an excess of rare alleles in VNI and VNBI. A) Two-way population comparisons of shared and fixed alleles between populations, and private alleles within each population of VNI, VNBI, and VNBII; B) Minor allele frequency in each of the VNI, VNBI and VNBII. VNI and VNBI had significantly more alleles than VNBII ($p<2.2\times10^{-16}$ for both comparisons, Fisher's exact test).
