# Supplementary material for: Population genomics and the evolution of virulence in the fungal pathogen Cryptococcus neoformans
Source: Genome Res. 2017 Jul;27(7):1207–19. doi: 10.1101/gr.218727.116 (PMC5495072; doi:10.1101/gr.218727.116)
Supplement: Supplemental Material [file supp_gr.218727.116_Supplemental_Fig_S4.pdf]

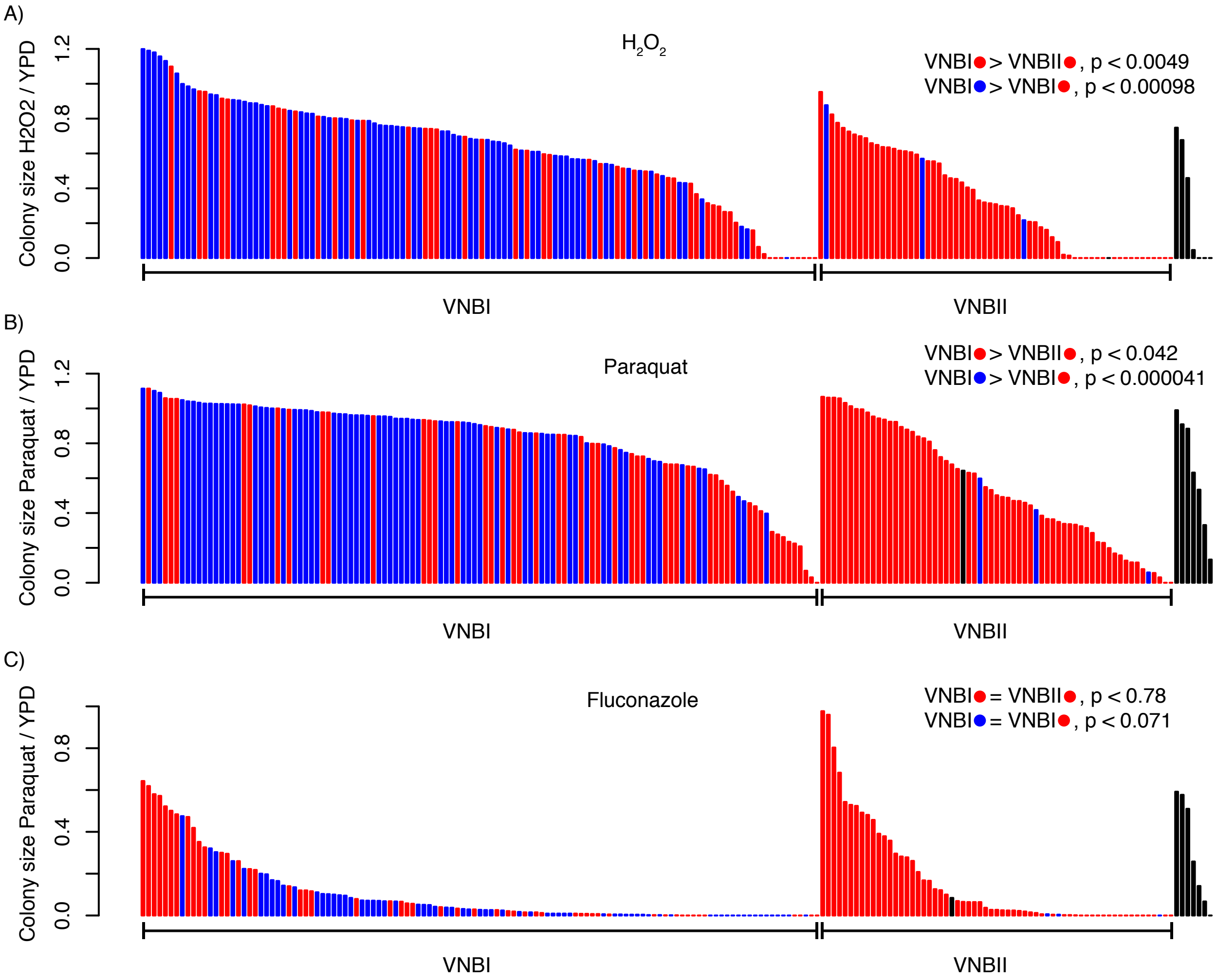

Supplemental Fig S4. Phenotypic analysis demonstrates that VNBI isolates are more resistant to oxidative stress than VNBII isolates. Isolates were challenged against two oxidative stressors (H2O2 and paraquat) and the antifungal fluconazole. Data is plotted as median colony size on the stressor over median colony size on YPD. Clinical isolates are shown in red, environmental isolates are shown in blue, and control strains are shown in black, and statistical comparisons are shown in the upper right of each plot. A) VNBI isolates were significantly more resistant to H_2O_2 than VNBII isolates, and VNBI environmental isolates were significantly more resistant to H_2O_2 than VNBI clinical isolates. B) VNBI isolates were significantly more resistant to paraquat than VNBII isolates, and VNBI environmental isolates were significantly more resistant to paraquat than VNBI clinical isolates. C) VNBII isolates had similar resistance to fluconazole as VNBI isolates, and VNBI clinical isolates had similar resistance to fluconazole as VNBI environmental isolates.
