# Supplementary material for: Population genomics and the evolution of virulence in the fungal pathogen Cryptococcus neoformans
Source: Genome Res. 2017 Jul;27(7):1207–19. doi: 10.1101/gr.218727.116 (PMC5495072; doi:10.1101/gr.218727.116)
Supplement: Supplemental Material [file supp_gr.218727.116_Supplemental_Fig_S5.pdf]

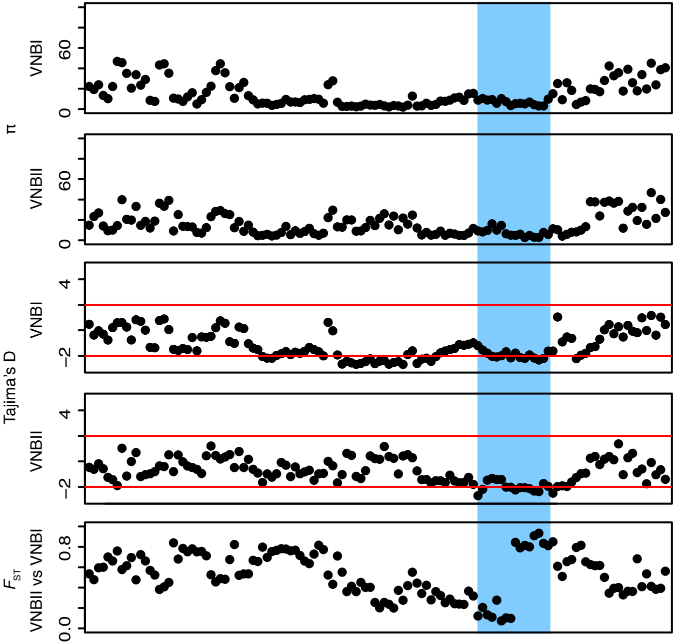

low F_{ST}
CNAG_07742 phosphatidylglycerol transfer protein
CNAG_07743 hypothetical protein
CNAG_01065 hypothetical protein
CNAG_01064 hypothetical protein
CNAG_01063 TOR protein KOG1

high F_{ST}
CNAG_01061 S/T protein kinase
CNAG_01060 hypothetical protein
CNAG_01059 vacuolar membrane protein
CNAG_01058 nitric oxide synthase-interacting protein
CNAG_01057 COP9 signalosome complex subunit 2

in between F_{ST}
CNAG_01062 CAMK/CAMKL/PASK protein kinase

Supplemental Fig S5. Region of chromosome 5 with abrupt change in FST between VNBI and VNBII. Genes in the low FST region, high FST region, and between the two regions are listed. The low FST region is the only region in the genome where loss of genetic diversity and a low Tajima's D is accompanied by a reduction in FST between two populations.
