# Supplementary material for: Population genomics and the evolution of virulence in the fungal pathogen Cryptococcus neoformans
Source: Genome Res. 2017 Jul;27(7):1207–19. doi: 10.1101/gr.218727.116 (PMC5495072; doi:10.1101/gr.218727.116)
Supplement: Supplemental Material [file supp_gr.218727.116_Supplemental_Fig_S6.pdf]

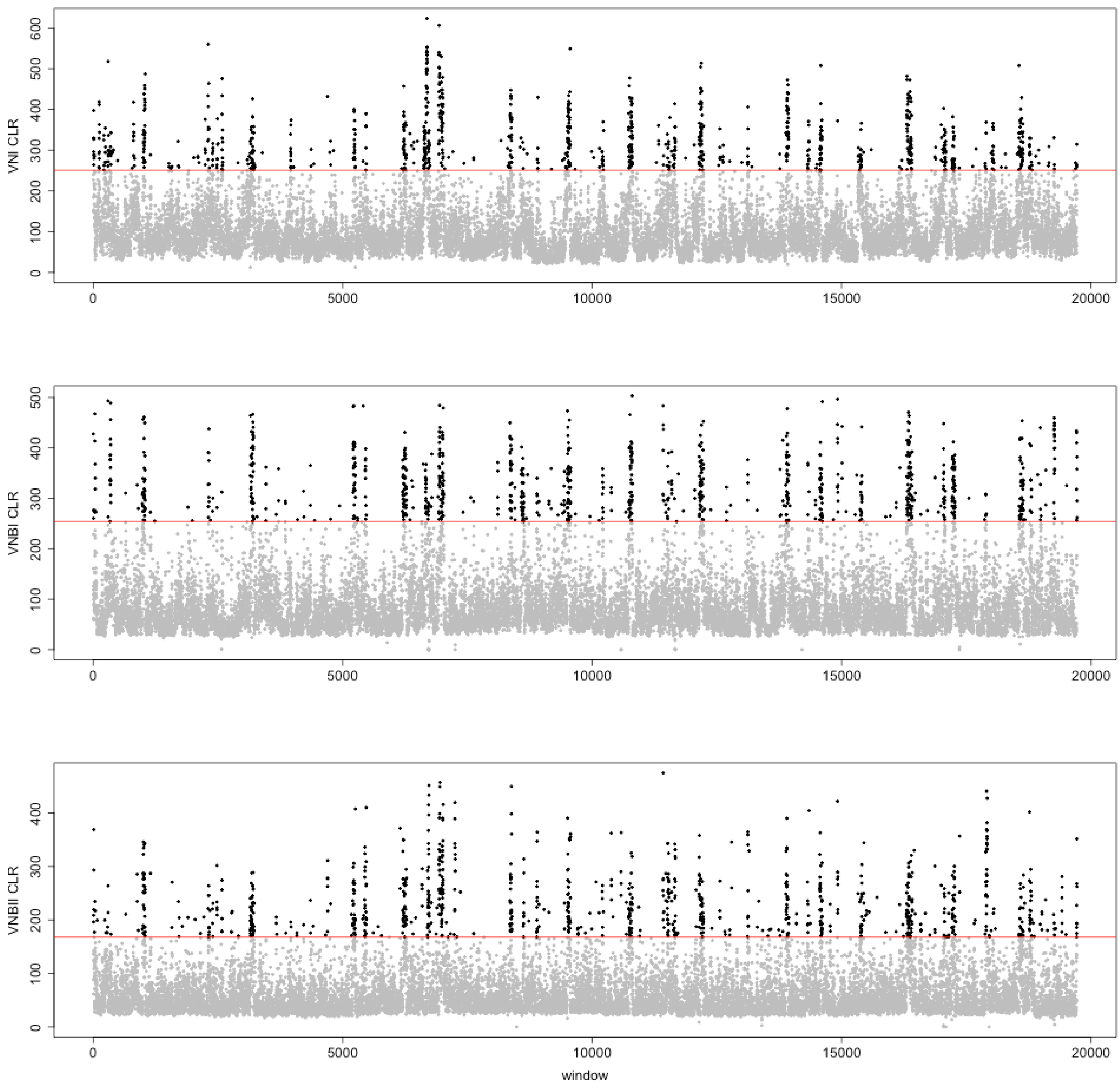


**Supplemental Fig S6.** Genome-wide signatures of selection across each of lineages VNI, VNBI, and VNBII. Values were computed using the composite likelihood ratio (CLR) test across windows of 50 segregating sites. Red lines indicate the top 5% of CLR values for each lineage; points above those lines are shown in black, while points below those lines are shown in grey.
