# Supplementary material for: Population genomics and the evolution of virulence in the fungal pathogen Cryptococcus neoformans
Source: Genome Res. 2017 Jul;27(7):1207–19. doi: 10.1101/gr.218727.116 (PMC5495072; doi:10.1101/gr.218727.116)
Supplement: Supplemental Material [file supp_gr.218727.116_Supplemental_Fig_S7.pdf]

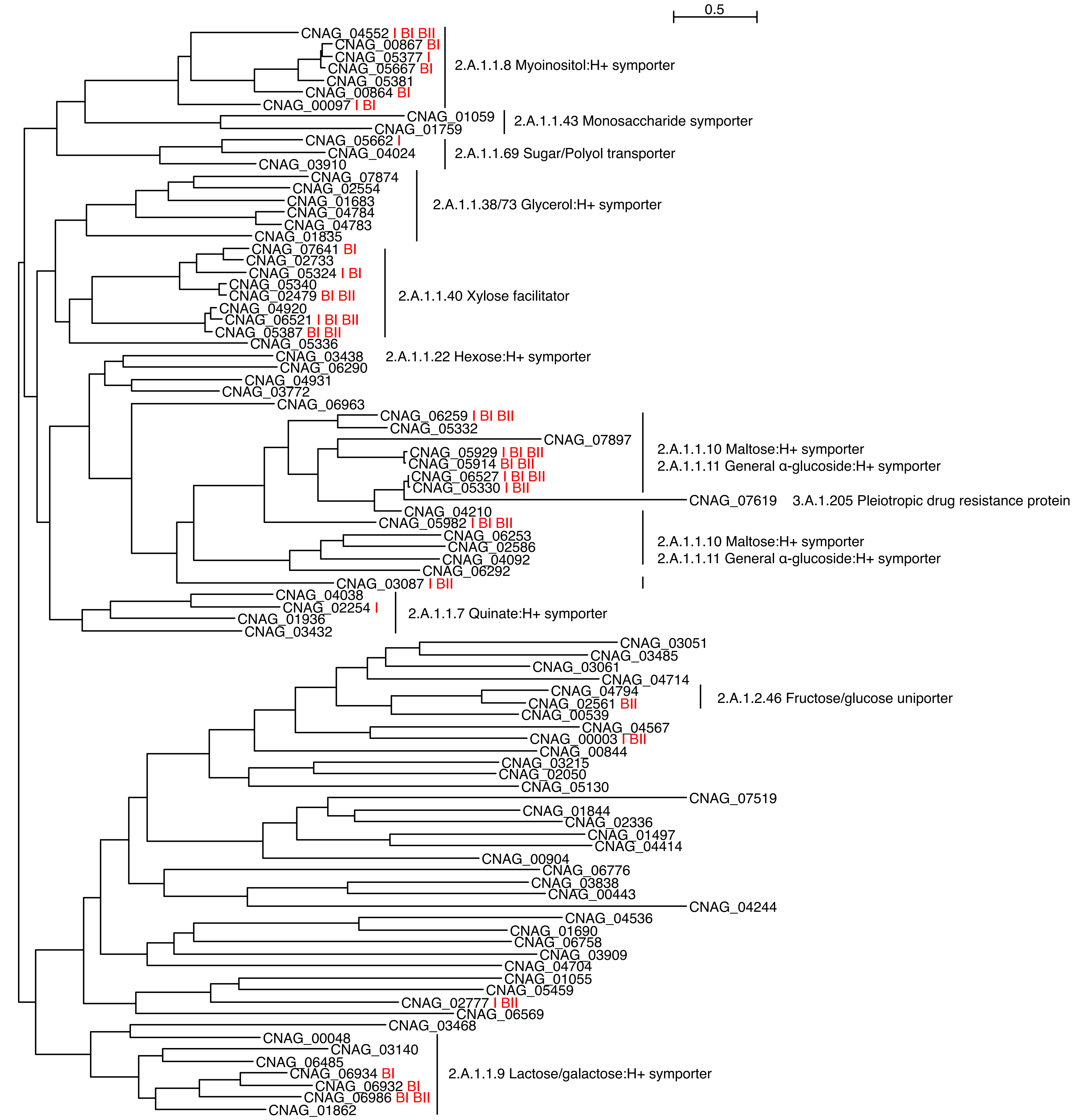

Supplemental Fig S7. Phylogenetic analysis of sugar transporters in the reference strain H99. Genes under selection in each lineage are labeled in red. Genes were annotated by best BLAST hits to the transporter classification database (http://www.tcdb.org/).
