# Supplementary material for: Population genomics and the evolution of virulence in the fungal pathogen Cryptococcus neoformans
Source: Genome Res. 2017 Jul;27(7):1207–19. doi: 10.1101/gr.218727.116 (PMC5495072; doi:10.1101/gr.218727.116)
Supplement: Supplemental Material [file supp_gr.218727.116_Supplemental_Fig_S8.pdf]

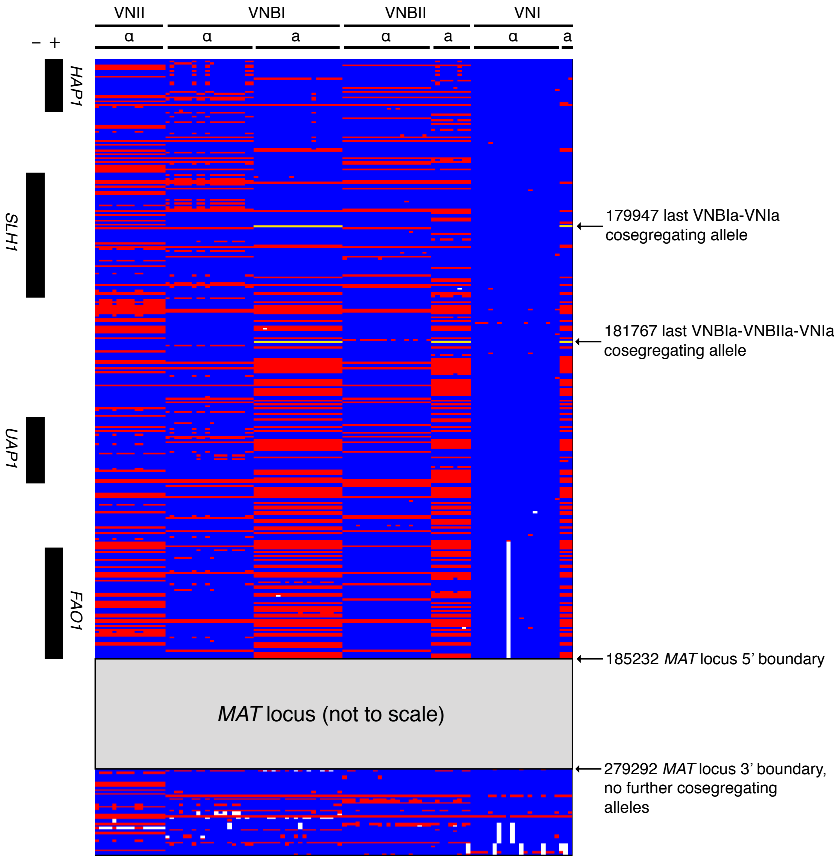

Supplemental Fig S8. Haplotype structure at the 5’ and 3’ boundaries of the *MAT* locus. Representative of VNI, VNBI, VNBII, VNII, including both mating types, are shown in columns. Rows represent variant positions; blue blocks represent reference genotypes, red blocks represent alternate genotypes, and white block represent ambiguous genotypes. Genes on the minus and plus strands are shown to the left of the blocks. At the 5’ end, the last co-segregating *MAT***a** allele of all three groups appears at 181767, while the last co-segregating *MAT***a** allele of VNBI and VNI appears at 179947, supporting the hypothesis that the VNBI and VNI are more closely related to each other than they are to VNBII.
