# Supplementary material for: Population genomics and the evolution of virulence in the fungal pathogen Cryptococcus neoformans
Source: Genome Res. 2017 Jul;27(7):1207–19. doi: 10.1101/gr.218727.116 (PMC5495072; doi:10.1101/gr.218727.116)
Supplement: Supplemental Material [file supp_gr.218727.116_Supplemental_Methods.docx]

**Supplemental Methods**

**Sample Selection**

The majority of isolates, particularly VNB isolates, were collected in Botswana and came from five localities: 1) clinical samples from Princess Marina Hospital, a major referral hospital in southern Botswana; 2) clinical samples from Nyangabgwe Referral Hospital, a referral hospital in northeastern Botswana; 3) from trees in the vicinity of Gaborone, where the Princess Marina is located; 4) from trees in the vicinity of Francistown, where Nyangabgwe Referral Hospital is located; and 5) from trees in the vicinity of Maun, in north-central Botswana. See (Chen et al. 2015) for more details.

**Sample Preparation**

Strains were grown overnight in 10 ml YPD at 30°C in shaking incubator (225 rpm). The cell pellets were processed for genomic DNA isolation using the MasterPure Yeast DNA Purification Kit (Epicentre, Madison, WI). The cells were incubated for 1 hour at 65°C in the cell lysis solution and then were treated twice with 5 µl 5 µg/µl RNase A. A lypophilization step was included for extraction of genomic DNA from cell pellets that were loose. The dried pellets were then pulverized by vortexing in a tube containing 3 mm glass beads.

**Variant Identification**

Variants were then identified using GATK version 3.4 (McKenna et al. 2010) as described. Briefly, indels were locally realigned, haplotypeCaller was invoked in GVCF mode with ploidy = 1, and genotypeGVCFs was used to predict variants in each strain. All VCFs were then combined and sites were filtered using variantFiltration with QD < 2.0, FS > 60.0, and MQ < 40.0. Individual genotypes were then filtered if the minimum genotype quality < 50, percent alternate allele < 0.8, or depth < 10.

**Population Genomic Analyses**

For analysis of VNI subdivisions, STRUCTURE was run with k=3, with 43% of the positions randomly subsampled from those containing at least 2 variants and missing in fewer than 5% of the strains (~94K sites); comparison to prior MLST analysis (Litvintseva et al. 2006) mapped AFLP genotypes to the three lineages as follows: VNIa corresponds to A2, A4, and A5, VNIb corresponds to A1 and A3, and VNIc was not assigned an AFLP type but corresponds to an African VNI clade in this prior study.

To identify smaller introgressions, we partitioned the marker data into 500 kb genome segments and repeated the STRUCTURE analysis using the same parameters. Segments showing incomplete population separation were excluded from the analysis. For each strain, we calculated the fraction of total marker sites with an alternate population fraction ≥ 0.9 in 5 kb windows. We identified those strains containing at least ten windows (50 kb total) across the genome exhibiting ≥ 75% markers showing an alternate population, and plotted the ancestry of all windows for those strains.

Linkage disequilibrium was calculated in 500 bp windows of all chromosomes except 5 (where the mating type locus is located) with vcftools version 1.14 (Danecek et al. 2011), using the --hap-r2 option with a minimum minor allele frequency of 0.1. Sliding-window population genetic statistics (π, Tajima’s D, F_ST_) were calculated in the PopGenome R package (Pfeifer et al. 2014) per chromosome in 5 kb windows. Genome-wide calculations used the average of all 5 kb windows. The CLR selection metric was also calculated using PopGenome with sliding windows of 50 segregating sites. The top 5% of windows were selected for each lineage. PFAM domains of genes within those regions were then compared to domains of the remaining genes using Fisher’s exact test corrected with the Benjamini-Hochberg method for multiple comparisons. For subtelomeric enrichment tests, the distribution of the top 5% of windows under selection between subtelomeric regions (40 kb from each chromosome end, as previously defined (Chow et al. 2012)) and the remainder of the chromosome was compared to a null distribution using Fisher’s exact tests.

**Phenotypic Assays**

Freezer stocks containing were streaked onto YPD petri dishes from either 50% glycerol or 1X Hogness buffer. Strains were grown at 30°C for two to five days. For each strain, a single colony was selected and added to 96 well microtiter plates containing 200 µL of YPD broth. Strain location in all plates was randomized to avoid position effects. Each 96 well plate contained six control strains (H99, *LAC1*, *MPK1*, *CNA1*, *RAS1*, and *HOG1*) and a YPD control. The 96 well plates were incubated for one to two days at 30°C. Colonies were pin replicated into 384 well microtiter plates containing 80 µL of YPD broth in each well. The 384 well plates were incubated for one to two days at 30C. They were then pinned onto one well solid agar plates in duplicate using the BM5-BC Colony Processing Robot (S & P Robotics) in 1536 array format.

**References**

Chen Y, Litvintseva AP, Frazzitta AE, Haverkamp MR, Wang L, Fang C, Muthoga C, Mitchell TG, Perfect JR. 2015. Comparative analyses of clinical and environmental populations of *Cryptococcus neoformans* in Botswana. *Mol Ecol* **24**: 3559–3571.

Chow EWL, Morrow CA, Djordjevic JT, Wood IA, Fraser JA. 2012. Microevolution of *Cryptococcus neoformans* driven by massive tandem gene amplification. *Mol Biol Evol* **29**: 1987–2000.

Danecek P, Auton A, Abecasis G, Albers CA, Banks E, DePristo MA, Handsaker RE, Lunter G, Marth GT, Sherry ST, et al. 2011. The variant call format and VCFtools. *Bioinformatics* **27**: 2156–2158.

Litvintseva AP, Thakur R, Vilgalys R, Mitchell TG. 2006. Multilocus sequence typing reveals three genetic subpopulations of *Cryptococcus neoformans* var. *grubii* (Serotype A), including a unique population in Botswana. *Genetics* **172**: 2223–2238.

McKenna A, Hanna M, Banks E, Sivachenko A, Cibulskis K, Kernytsky A, Garimella K, Altshuler D, Gabriel S, Daly M, et al. 2010. The Genome Analysis Toolkit: a MapReduce framework for analyzing next-generation DNA sequencing data. *Genome Res* **20**: 1297–1303.

Pfeifer B, Wittelsbürger U, Ramos-Onsins SE, Lercher MJ. 2014. PopGenome: an efficient swiss army knife for population genomic analyses in R. *Mol Biol Evol* **31**: 1929–1936.
