# Supplementary material for: Population genomics and the evolution of virulence in the fungal pathogen Cryptococcus neoformans
Source: Genome Res. 2017 Jul;27(7):1207–19. doi: 10.1101/gr.218727.116 (PMC5495072; doi:10.1101/gr.218727.116)
Supplement: Supplemental Material [file supp_gr.218727.116_Supplemental_Table_S2.docx]

**Supplemental Table S2.** Gains and losses of functionally annotated genes in VNBI and VNBII. All genes other than those annotated as hypothetical proteins are listed. Representative genes belong to Tu401 (VNBI) and Ze90 (VNBII).

| Category | Gene annotation | Representative gene |
| --- | --- | --- |
| VNBI Gains | α-β hydrolase | C366_00539 |
| VNBI Losses | phosphopyruvate hydratase | C367_02389 |
|  | alginate lyase | C367_02222 |
| VNBII Gains | L-iditol 2-dehydrogenase | C367_04669 |
| VNBII Losses | nuclear protein | C366_04628 |
