# Supplementary material for: Population genomics and the evolution of virulence in the fungal pathogen Cryptococcus neoformans
Source: Genome Res. 2017 Jul;27(7):1207–19. doi: 10.1101/gr.218727.116 (PMC5495072; doi:10.1101/gr.218727.116)
Supplement: Supplemental Material [file supp_gr.218727.116_Supplemental_Table_S3.docx]

**Supplemental Table S3.** Enrichment of VNBI versus VNBII isolates in geographic locations. For each collection locality, the region within Botswana is listed, along with the isolation source and counts of VNBI and VNBII strains. VNBII strains were significantly enriched in the Princess Marina Hospital isolates relative to Nyangabgwe Referral Hospital, Francistown and Maun isolates (Fisher’s exact test, p < 3.3e^-8^, 4.9e^-14^, and 1.8e^-7^, respectively).

| Location | Region | Source | VNBI | VNBII |
| --- | --- | --- | --- | --- |
| Princess Marina Hospital | Southeast | Clinical | 22 | 51 |
| Gaborone | Southeast | Environmental | 1 | 0 |
| Nyangabgwe Referral Hospital | Northeast | Clinical | 26 | 3 |
| Francistown | Northeast | Environmental | 41 | 0 |
| Maun | North-Central | Environmental | 21 | 2 |
