# Supplementary material for: Population genomics and the evolution of virulence in the fungal pathogen Cryptococcus neoformans
Source: Genome Res. 2017 Jul;27(7):1207–19. doi: 10.1101/gr.218727.116 (PMC5495072; doi:10.1101/gr.218727.116)
Supplement: Supplemental Material [file supp_gr.218727.116_Supplemental_Table_S4.docx]

**Supplemental Table S4.** VNBI populations are mixed across geographic locations and isolation sources. For each isolate from a given location, the most genetically similar isolate not from the same location was identified, and the results were tabulated below. For most localities, there were no significant genetic associations between geographies, except for Maun, where isolates were significantly more likely to be closest to isolates from Nyangabgwe Referral Hospital or Francistown than Princess Marina Hospital. These results suggest a large, well-mixed population with frequent transitions from environment to clinic.

| Location | PMH | FTN | MAU | NRH | P-value |
| --- | --- | --- | --- | --- | --- |
| Princess Marina Hospital | -- | 10 | 9 | 3 | 0.1018 |
| Francistown | 7 | -- | 17 | 17 | 0.1036 |
| Maun | 1 | 8 | -- | 12 | 0.0091* |
| Nyangabgwe Referral Hospital | 6 | 16 | 4 | -- | 0.3842 |
