# Supplementary material for: Population genomics and the evolution of virulence in the fungal pathogen Cryptococcus neoformans
Source: Genome Res. 2017 Jul;27(7):1207–19. doi: 10.1101/gr.218727.116 (PMC5495072; doi:10.1101/gr.218727.116)
Supplement: Supplemental Material [file supp_gr.218727.116_Supplemental_Table_S6.docx]

**Supplemental Table S6.** GWAS analysis reveals genes and intergenic regions associated with the increased melanization in VNBI and VNBII. Two GWAS analyses were conducted. In the first, variants under 5% frequency were combined by gene or intergenic region (rare) while variants over 5% frequency were treated independently (common). In the second analysis, loss-of-function mutations were identified and combined by gene (LOF). Both analyses were conducted using GEMMA corrected for population stratification with a relatedness matrix. The 10 most significant features across both analyses are shown.

| P value | Hit Type | Feature | Genes(s) |
| --- | --- | --- | --- |
| 3.43×10^-9^ | LOF | CNAG_03346 | *BZP4* |
| 4.39×10^-9^ | common | CNAG_01996 | hypothetical protein |
| 2.53×10^-8^ | common | intergenic: CNAG_06251-CNAG_06252 | Ser/Thr protein phosphatase family protein;  hypothetical protein |
| 2.53×10^-8^ | common | CNAG_07009 | hypothetical protein |
| 2.85×10^-8^ | common | intergenic: CNAG_06250- CNAG_06251 | hypothetical protein; Ser/Thr protein phosphatase family protein |
| 2.85×10^-8^ | common | CNAG_06251 | Ser/Thr protein phosphatase family protein |
| 4.75×10^-8^ | common | intergenic: CNAG_04004-CNAG_04005 | small subunit ribosomal protein S1;  cytoplasmic protein, negative regulator of differentiation |
| 4.75×10^-8^ | common | intergenic: CNAG_04005-CNAG_04006 | cytoplasmic protein, negative regulator of differentiation; hypothetical protein |
| 5.18×10^-8^ | rare | CNAG_04357 | hypothetical protein |
| 7.33×10^-8^ | common | intergenic: CNAG_07506-CNAG_07507 | transcriptional repressor NF-X1; protein kinase regulator |
