# Supplementary material for: Population genomics and the evolution of virulence in the fungal pathogen Cryptococcus neoformans
Source: Genome Res. 2017 Jul;27(7):1207–19. doi: 10.1101/gr.218727.116 (PMC5495072; doi:10.1101/gr.218727.116)
Supplement: Supplemental Material [file supp_gr.218727.116_Supplemental_Table_S7.docx]

**Supplemental Table S7.** GWAS analysis reveals genes and intergenic regions associated with the increased resistance to hydrogen peroxide in VNBI and VNBII. Two GWAS analyses were conducted. In the first, variants under 5% frequency were combined by gene or intergenic region (rare) while variants over 5% frequency were treated independently (common). In the second analysis, loss-of-function mutations were identified and combined by gene (LOF). Both analyses were conducted using GEMMA corrected for population stratification with a relatedness matrix. The 10 most significant features across both analyses are shown.

| P value | Hit Type | Feature | Genes(s) |
| --- | --- | --- | --- |
| 6.73×10^-6^ | common | CNAG_00167 | hypothetical protein |
| 1.34×10^-5^ | common | CNAG_02566 | hepatocyte nuclear factor |
| 6.48×10^-5^ | common | CNAG_04139 | hypothetical protein |
| 6.57×10^-5^ | common | intergenic: CNAG_06772- CNAG_06771 | nucleoporin *SEH1*; hypothetical protein |
| 7.64×10^-5^ | rare | CNAG_06815 | hypothetical protein |
| 1.29×10^-4^ | rare | CNAG_02912 | hypothetical protein |
| 1.76×10^-4^ | common | CNAG_07314 | hypothetical protein |
| 2.38×10^-4^ | common | intergenic: CNAG_04139-CNAG_04140 | hypothetical protein; hypothetical protein |
| 2.59×10^-4^ | common | intergenic: CNAG_07560-CNAG_07561 | hypothetical protein; 6-phosphogluconate dehydrogenase, decarboxylating 1 |
| 2.61×10^-4^ | common | CNAG_07757 | hypothetical protein |
