# Supplementary material for: Population genomics and the evolution of virulence in the fungal pathogen Cryptococcus neoformans
Source: Genome Res. 2017 Jul;27(7):1207–19. doi: 10.1101/gr.218727.116 (PMC5495072; doi:10.1101/gr.218727.116)
Supplement: Supplemental Material [file supp_gr.218727.116_Supplemental_Table_S8.docx]

**Supplemental Table S8.** GWAS analysis reveals genes and intergenic regions associated with the increased resistance to paraquat in VNBI and VNBII. Two GWAS analyses were conducted. In the first, variants under 5% frequency were combined by gene or intergenic region (rare) while variants over 5% frequency were treated independently (common). In the second analysis, loss-of-function mutations were identified and combined by gene (LOF). Both analyses were conducted using GEMMA corrected for population stratification with a relatedness matrix. The 10 most significant features across both analyses are shown.

| P value | Hit Type | Feature | Genes(s) |
| --- | --- | --- | --- |
| 1.33×10^-5^ | rare | intergenic: CNAG_04676- CNAG_04675 | 6-phosphofructokinase; hypothetical protein |
| 1.98×10^-5^ | common | CNAG_03061 | multiple drug resistance protein |
| 3.00×10^-5^ | common | CNAG_02157 | hypothetical protein |
| 3.00×10^-5^ | common | intergenic: CNAG_02157- CNAG_02156 | hypothetical protein; hypothetical protein |
| 3.40×10^-5^ | common | intergenic: CNAG_03057- CNAG_03056 | hypothetical protein; hypothetical protein |
| 3.40×10^-5^ | common | CNAG_03054 | hypothetical protein |
| 3.40×10^-5^ | common | intergenic: CNAG_03052-CNAG_03051 | PP2Cc protein phosphatase; polyamine transporter |
| 3.40×10^-5^ | common | CNAG_03051 | polyamine transporter |
| 3.57×10^-5^ | common | intergenic: CNAG_04587-CNAG_04586 | hypothetical protein; LIM-homeobox protein |
| 4.44×10^-5^ | common | CNAG_04586 | LIM-homeobox protein |
