# Supplementary material for: Population genomics and the evolution of virulence in the fungal pathogen Cryptococcus neoformans
Source: Genome Res. 2017 Jul;27(7):1207–19. doi: 10.1101/gr.218727.116 (PMC5495072; doi:10.1101/gr.218727.116)
Supplement: Supplemental Material [file supp_gr.218727.116_Supplemental_Table_S9.docx]

**Supplemental Table S9.** GWAS analysis reveals genes and intergenic regions associated with the increased resistance to fluconazole in VNBI and VNBII. Two GWAS analyses were conducted. In the first, variants under 5% frequency were combined by gene or intergenic region (rare) while variants over 5% frequency were treated independently (common). In the second analysis, loss-of-function mutations were identified and combined by gene (LOF). Both analyses were conducted using GEMMA corrected for population stratification with a relatedness matrix. The 10 most significant features across both analyses are shown.

| P value | Hit Type | Feature | Genes(s) |
| --- | --- | --- | --- |
| 2.26×10^-7^ | common | intergenic: CNAG_07877- CNAG_05455 | hypothetical protein; translation initiation factor eIF-1A |
| 1.57×10^-6^ | LOF | CNAG_04786 | hypothetical factor |
| 2.44×10^-6^ | rare | CNAG_06102 | ADP/ATP carrier protein |
| 3.38×10^-6^ | common | CNAG_01559 | hypothetical protein |
| 3.38×10^-6^ | common | intergenic: CNAG_01559- CNAG_01560 | hypothetical protein; hypothetical protein |
| 3.38×10^-6^ | common | intergenic: CNAG_01561- CNAG_01562 | WD-repeat protein; hypothetical protein |
| 7.51×10^-6^ | common | intergenic: CNAG_05344- CNAG_05345 | hemolysin; amino acid transporter |
| 1.07×10^-5^ | common | intergenic: CNAG_05343-CNAG_05344 | hypothetical protein; hemolysin |
| 1.14×10^-5^ | common | intergenic: CNAG_05346-CNAG_07869 | sarcosine oxidase; hypothetical protein |
| 1.38×10^-5^ | common | intergenic: CNAG_01557- CNAG_01558 | calmodulin; NAD-dependent (R,R)-butanediol dehydrogenase |
