# Supplementary material for: Population genomics and the evolution of virulence in the fungal pathogen Cryptococcus neoformans
Source: Genome Res. 2017 Jul;27(7):1207–19. doi: 10.1101/gr.218727.116 (PMC5495072; doi:10.1101/gr.218727.116)
Supplement: Supplemental Material [file supp_gr.218727.116_Supplemental_Table_S10.docx]

**Supplemental Table S10.** Enriched functions in regions under selection identified with the composite likelihood ratio (CLR) test. CLR was calculated for sliding windows of 50 segregating sites, and the top 5% of windows were selected for each lineage. PFAM domains of genes within those regions were then compared to domains of the remaining genes using Fisher’s exact test corrected with the Benjamini-Hochberg method for multiple comparisons.

| PFAM domain | VNI | VNBI | VNBII |
| --- | --- | --- | --- |
| PF00083 Sugar transporter | 0.0011 | 2.8×10^-5^ | 0.0058 |
| PF07690 MFS transporter | 0.015 | 0.021 | 0.0034 |
| PF02894 Oxidoreductase family, C-terminal alpha/beta domain | 0.029 | - | - |
| PF13738 Pyridine nucleotide-disulphide oxidoreductase | 0.042 | - | - |
| PF13434 L-lysine 6-monooxygenase | 0.042 | - | - |
| PF07691 PA14 domain | - | 0.0074 | - |
| PF00933 Glycosyl hydrolase family 3 N terminal domain | - | 0.021 | - |
| PF01915 Glycosyl hydrolase family 3 C-terminal domain | - | 0.021 | - |
| PF14310 Fibronectin type III-like domain | - | 0.021 | - |
