# Supplementary material for: Population genomics and the evolution of virulence in the fungal pathogen Cryptococcus neoformans
Source: Genome Res. 2017 Jul;27(7):1207–19. doi: 10.1101/gr.218727.116 (PMC5495072; doi:10.1101/gr.218727.116)
Supplement: Supplemental Material [file supp_gr.218727.116_Supplemental_Table_S11.docx]

**Supplemental Table S11.** Enrichment of regions under selection in sub-telomeric regions. The number of windows under selection in the subtelomeric 40 Kb ends of each chromosome were compared to the expected distribution using Fisher’s exact test. Significant p-values for each lineage and non-significant values are indicated as NS. Subtelomeric regions were enriched for areas under selection in 8 chromosomes in VNI, 5 chromosomes in VNBI, and 10 chromosomes in VNBII.

| Chromosome | P-values | | |
| --- | --- | --- | --- |
|  | **VNI** | **VNBI** | **VNBII** |
| 1 | 8.6×10^-5^ | 0.0026 | 0.0046 |
| 2 | NS | NS | NS |
| 3 | NS | NS | 0.014 |
| 4 | 2.8×10^-8^ | 0.0094 | 0.034 |
| 5 | 0.012 | 7.6×10^-5^ | 1.2×10^-5^ |
| 6 | NS | NS | NS |
| 7 | 0.018 | NS | NS |
| 8 | NS | NS | 0.0042 |
| 9 | 0.040 | NS | 0.0036 |
| 10 | 8.4×10^-5^ | NS | NS |
| 11 | NS | 0.0040 | 0.063 |
| 12 | 5.0×10^-5^ | NS | 0.030 |
| 13 | NS | NS | 8.5×10^-7^ |
| 14 | 0.0072 | 0.0027 | 4.3×10^-4^ |
