# Supplementary material for: Population genomics and the evolution of virulence in the fungal pathogen Cryptococcus neoformans
Source: Genome Res. 2017 Jul;27(7):1207–19. doi: 10.1101/gr.218727.116 (PMC5495072; doi:10.1101/gr.218727.116)
Supplement: Supplemental Material [file supp_gr.218727.116_Supplemental_Table_S12.docx]

**Supplemental Table S12.** Enriched functions in subtelomeric regions under selection identified with the composite likelihood ratio (CLR) test. CLR was calculated for sliding windows of 50 segregating sites, and the top 5% of windows were selected for each lineage. Windows in the first and last 40 Kb of each chromosome were considered subtelomeric. PFAM domains of genes within those regions were then compared to domains of the remaining genes using Fisher’s exact test corrected with the Benjamini-Hochberg method for multiple comparisons.

| PFAM domain | VNI | VNBI | VNBII |
| --- | --- | --- | --- |
| PF00083 Sugar transporter | 1.7×10^-7^ | 6.0×10^-9^ | 4.3×10^-5^ |
| PF07690 MFS transporter | 1.2×10^-8^ | 1.4×10^-4^ | 3.0×10^-5^ |
| PF04082 Fungal specific transcription factor | 0.0082 | 0.018 | 0.037 |
| PF02894 Oxidoreductase family, C-terminal alpha/beta domain | 0.0030 | - | - |
| PF13738 Pyridine nucleotide-disulphide oxidoreductase | - | - | - |
| PF13434 L-lysine 6-monooxygenase | 0.020 | - | - |
| PF00743 Flavin-binding monooxygenase-like | 0.030 | - | - |
| PF01408 Oxidoreductase family, NAD-binding Rossmann fold | 0.0030 | - | - |
| PF07691 PA14 domain | - | 0.0042 | - |
| PF00933 Glycosyl hydrolase family 3 N terminal domain | - | 0.0083 | - |
| PF01915 Glycosyl hydrolase family 3 C-terminal domain | - | 0.0083 | - |
| PF14310 Fibronectin type III-like domain | - | 0.0084 | - |
| PF05721 Phytanoyl-CoA dioxygenase | - | 0.017 | - |
