# Supplementary material for: Population genomics and the evolution of virulence in the fungal pathogen Cryptococcus neoformans
Source: Genome Res. 2017 Jul;27(7):1207–19. doi: 10.1101/gr.218727.116 (PMC5495072; doi:10.1101/gr.218727.116)
Supplement: Supplemental Material [file supp_gr.218727.116_Supplemental_Fig_S1.pdf]

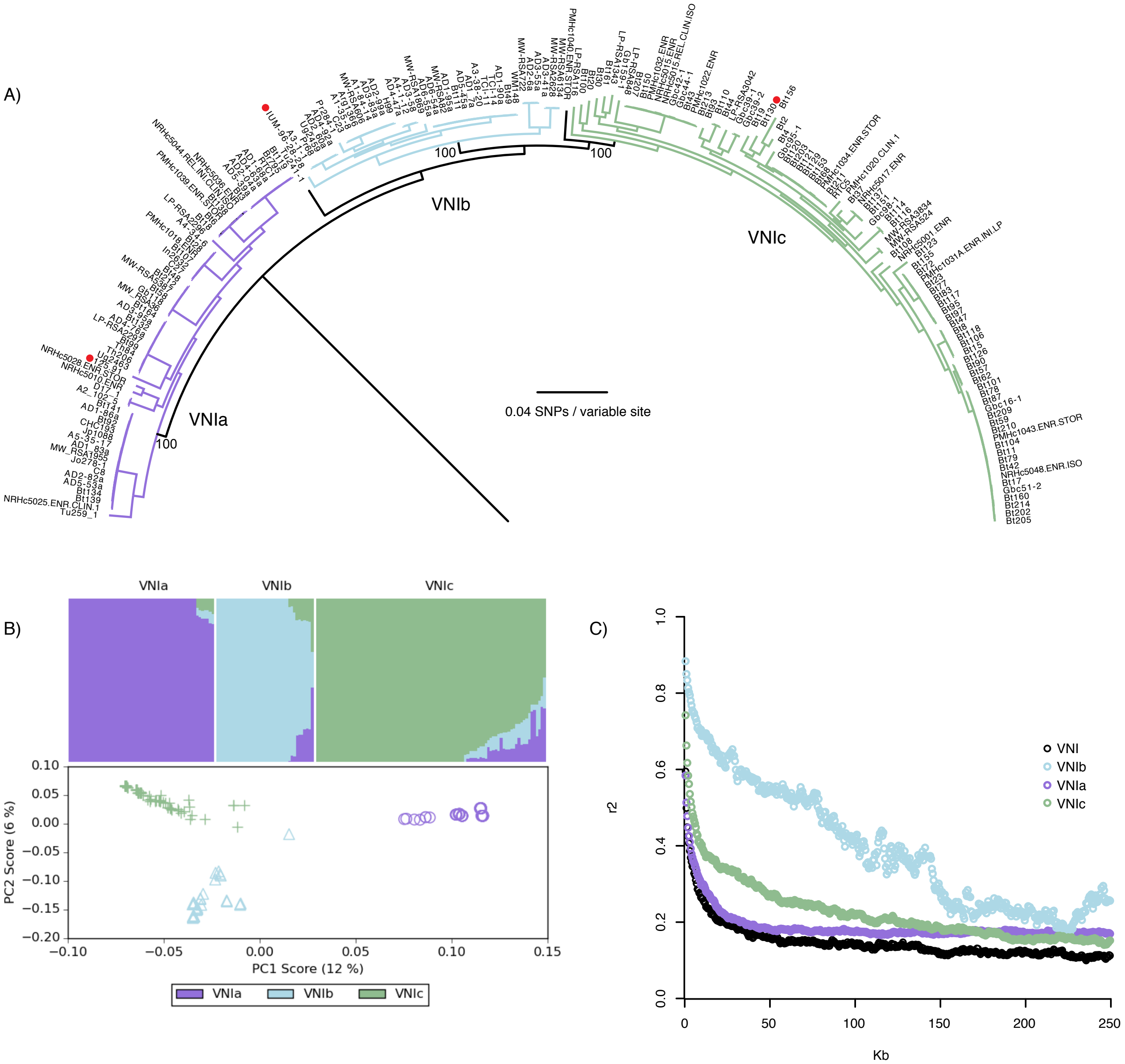

Supplemental Fig S1. VNI is comprised of three recombining sub-clades. A) Phylogenetic analysis divides VNI into three major sub-clades, the global VNIa and VNIb, and the southeast African VNIc; B) STRUCTURE and PCA analysis show largely distinct sub-populations with a small degree of mixing; C) Linkage decays more rapidly in the combined VNI dataset than any individual sub-clade, suggesting the sub-clades are currently recombining with each other and do not represent reproductively isolated populations.
